# Clinical Yield of Ileal Intubation During Screening Colonoscopy

**DOI:** 10.7759/cureus.20870

**Published:** 2022-01-02

**Authors:** Amer A Alkhatib, Shiva Kumar

**Affiliations:** 1 Gastroenterology and Hepatology, Cleveland Clinic Abu Dhabi, Abu Dhabi, ARE; 2 Gastroenterology and Hepatology, Cleveland Clinic Lerner College of Medicine, Cleveland, USA

**Keywords:** united arab emirates, arab countries, middle east, ileum, terminal ileum, colonoscopy, colonoscope, screening, screening colonoscopy, clinical diagnostic value

## Abstract

Objectives

Ileal intubation during screening colonoscopy can serve as supportive evidence of complete examination. However, most studies conducted in Western countries showed a limited value of ileal inspection in asymptomatic patients undergoing colonoscopy. Therefore, our aim is to determine the clinical yield of routine ileal examination during the performance of screening colonoscopy in a cohort of patients in the Middle East and identify factors associated with successful ileal intubation in this setting.

Methods

A retrospective review of a prospectively collected database of all screening colonoscopies was performed at a single endoscopy unit. The patients were divided into two groups; group A included patients in whom the extent of examination was the cecum and group B comprised of those who underwent ileal intubation as well. We summarized the endoscopic and pathological findings of the ileoscopic examinations and their clinical impact. Univariate and multivariate analyses were used to compare both groups and to identify factors predictive of ileal intubation in the setting of screening colonoscopy.

Results

Two thousand four hundred seventy-three unique completed screening colonoscopies were analyzed (group A=1465 patients, group B=1008 patients). Overall Ileal intubation rate was 40.8%. Of the patients in group B, 3.7% were noted to have findings on ileoscopy, which were deemed to be clinically significant in almost half (1.8% overall). Univariate analysis identified the following factors as being predictive of ileal intubation during screening colonoscopy: patients' age (51.7 vs. 53.5 years, p<0.001), short cecal insertion time, endoscopists' specialty (gastroenterology 42.3% vs. surgery 24.3%, p<0.001), type of colonoscope (pediatric 47.1% vs. adult 33.5% colonoscope, p<0.001), and quality of preparation in the right colon (poor vs. adequate/good : (25.6% vs. 42.5%, p<0.001). Mixed-effects logistic regression identified patients' age, endoscopist specialty, quality of right colon preparation, and cases with short insertion time as independent variables predicting ileal intubation during SC

Conclusion

The clinical yield of routine ileal intubation during screening colonoscopy is low. Ileal intubation during screening colonoscopy in our cohort was more likely in younger patients with adequate/good right colon preparation and when the exam is performed by a gastroenterologist, in cases with short insertion time. Prospective studies are needed to assess our research findings and to determine the clinical value of routinely intubating terminal ileum during screening colonoscopy in the population of the Middle East.

## Introduction

Ileal intubation is technically feasible in almost all patients undergoing colonoscopy [1-4]. It serves as a reliable confirmatory step of a complete colonoscopic examination [5]. However, the reported clinical yield of routine ileal examination during colonoscopy performed for other indications is variable depending on the studied cohorts, with the likelihood of endoscopic ileal findings ranging from 0.4% to 67% [4]. While there are numerous publications addressing the clinical significance of ileal intubation during colonoscopy for other indications, there are limited data evaluating the clinical significance of elective ileal inspection during routine screening colonoscopy (SC).

The reported phenotype of Crohn’s disease in the Middle East is different, with increasing disease prevalence [6,7]. Therefore, it is not clear whether the yield of routine ileal intubation during screening colonoscopy will be different compared to other studies that were conducted in Western countries.

Therefore, the aim of our study was to assess the clinical yield of routine ileal intubation in patients undergoing screening colonoscopy in the Middle East. Moreover, the study aims to identify factors associated with successful ileal intubation in this setting.

## Materials and methods

We conducted a retrospective review of a prospectively collected database of all lower gastrointestinal endoscopies that were performed at a single center from May 2015 to December 2018. All SCs performed in patients at average risk of colorectal neoplasia were included in the analysis. Data collected included patients’ age and gender, endoscopist specialty, quality of the colon preparation, total procedure, insertion and withdrawal times, maneuvers performed, endoscopic and histopathological findings. 

Furthermore, we compared those in whom the extent of the examination was the cecum (group A) to those who underwent ileoscopy as well (group B).

Point and interval estimates were reported for all descriptive data and presented as means ± the standard deviation. Regarding categorical variables, frequencies (n) and percentages (%) were used. The Student’s t-test was used to compare means and Pearson’s chi-square test to compare categorical variables. Univariate and multivariate analyses were utilized to identify variables predictive of ileal intubation during SC.

We used multivariate analysis to investigate the association between different variables and TII in patients undergoing SC. Because of clustering or lack of independence of the outcome (successful TII) at the endoscopist level, we used a mixed-effects logistic regression utilizing the command melogit in Stata 15.1 (StataCorp LP, College Station, Texas). Mixed-effects logistic regression provides a fixed effect estimate of the odds ratio for the association between type of colonoscope and successful TII, adjusting for the additional covariates, and accounting for clustering by including a random effect at the endoscopist level.

Statistical significance was defined as p-value<0.05. All statistical analyses were performed using the statistical package Stata version 15.1. The study protocol was approved by the institutional review board of Cleveland Clinic Abu Dhabi in the United Arab Emirates (UAE).

## Results

Eight thousand one hundred forty-seven colonoscopies were performed during the study period, of which 2473 were SCs in individuals at average risk of colorectal neoplasia. The patients were referred from their primary care providers, the surgery clinic, or the gastroenterology clinic for screening colonoscopy. The included cohort of patients reported no significant gastrointestinal symptoms. All patients received either MoviPrep® (Salix Pharmaceuticals, Bridgewater Township, New Jersey) or Picoprep® (Ferring Pharmaceuticals, Saint-Prex, Switzerland) for bowel preparation prior to colonoscopy.

**Figure 1:**
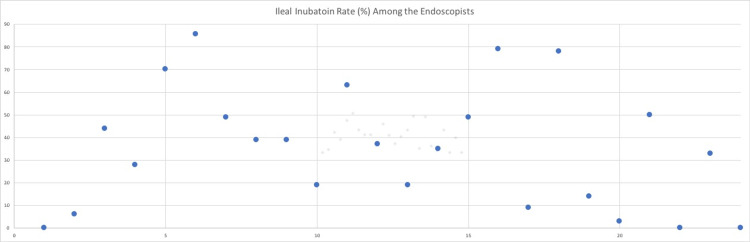
A scatter plot that illustrates variable ileal intubation rates % (y-axis) for each individual endoscopist (x-axis)

In 1465 colonoscopies, the reported extent of the examination was the cecum (group A). Ileal intubation was performed and reported in the remaining 1008 cases (group B), resulting in a 40.8% ileal intubation rate. However, there was a wide variation in the Ileal intubation rate among the endoscopists ranging from 0 to 85.7% (Figure 1).

The procedures were performed by 24 endoscopists (17 gastroenterologists and seven surgeons) from a wide range of diverse international training backgrounds. Among gastroenterologists, eight were trained in the USA, three in Europe, two in Canada, two in Asia, and one each in Africa and Australia. All surgeons performing endoscopy were trained in the USA, except one (Europe). Based on their endoscopic training, all endoscopists were credentialled to perform colonoscopy prior to joining our institution. 

In group A (n=1465), 55.1% were males, and 44.9% were females, with a mean age of 53.5± 9.7 years. The mean total procedure time was (20.1 ± 9.6 min, (IQR 13.8-23.6 min), with insertion and withdrawal times being (7.5± 5.5 min (IQR 4.1-9.2 min) and 11.8± 7.2 min (IQR 6.9-14.1 min) respectively. Adult colonoscopes were used in 52.1% of the cases, and pediatric colonoscopes in the remainder. 

In group B (n=1008), 54.3% were males, and 45.7% were females, with a mean age of 51.7± 9.9. The mean total procedure time was 19.7± 8.5 min (IQR 13.9-23.4 min), with insertion and withdrawal times being 5.6± 4.0 min (IQR 3-6.9) and 13.5± 6.9 mins (IQR 8.8-16.3) respectively. Adult colonoscopes were used in 385 cases (38.2%), and pediatric colonoscopes in the remainder.

Table 1 summarizes the clinical and demographic characteristics of both study groups.

**Table 1:** Patients and procedural characteristics of the study population The listed values are mean ± (standard deviation) for continuous variables and % (n) for categorical variables. * 25 missing data for the type of sedation

	Patients in whom cecum was reached but ileocecal valve was not intubated (group A)	Patients in whom ileocecal valve was intubated (group B)	p-value
Number of cases	1465	1008	
Males	55.1% (807)	54.3% (547)	0.687
Females	44.9% (658)	45.7% (461)	0.687
Age in years	53.5 (9.7)	51.7 (9.9)	<0.001
Young (≤ 65-year-old)	88.4% (1295)	91.0% (917)	0.040
Geriatric (> 65-year-old)	11.6% (170)	9.0% (91)	0.040
Conscious sedation	5.0% (73)*	4.3% (43)*	0.396
Monitored anesthesia care	95.0% (1375)*	95.7% (957)*	0.396
Pediatric colonoscope	47.9% (701)	61.8% (623)	<0.001
Adult colonoscope	52.1% (764)	38.2% (385)	<0.001
Total procedure time (min)	20.1 (9.6)	19.7 (8.5)	0.370
Insertion time (min)	7.5 (5.5)	5.6 (4.0)	<0.001
Withdrawal time (min)	11.8 (7.2)	13.5 (6.9)	<0.001
Poor preparation of the entire colon or the right colon	12.9% (189)	6.5% (65)	<0.001
Performed by gastroenterologists	89.4% (1309)	95.0% (958)	<0.001
Performed by surgeons	10.6% (156)	5.0 % (50)	<0.001

**Figure 2:**
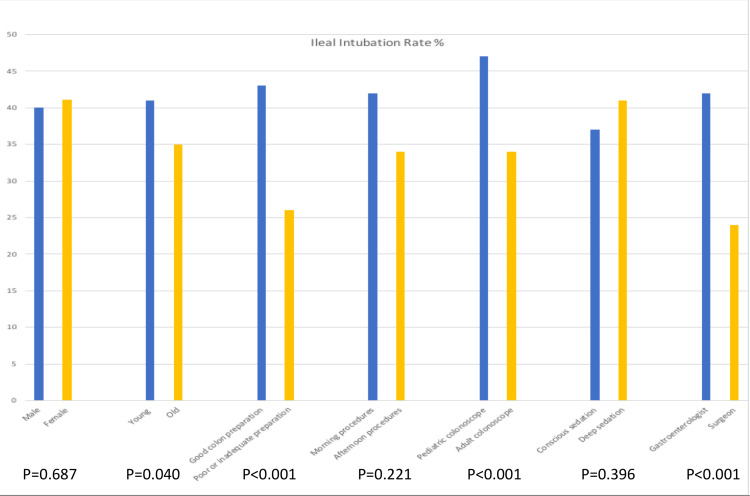
Terminal ileum intubation rate based on variable factors

Univariate analysis identified four factors that were statistically associated with ileal intubation during screening colonoscopy: endoscopists’ specialty (gastroenterology 42.3% vs. surgery 24.3%, p<0.001), type of colonoscope (pediatric colonoscope 47.1% vs. adult colonoscope 33.5%, p<0.001), younger patients’ age, and quality of the preparation in the right colon (poor 25.6% vs. adequate/good 42.5%, P<0.001). Figure 2 illustrates different ileal intubation rates based on variable factors that are patient, physician, and procedure-related.

In multivariable analysis, mixed-effects logistic regression of the available data yielded a significant association between achieving ileal intubation and younger age of the patients, good colon preparation, shorter cecal insertion time, and procedures performed by gastroenterologists rather than surgeons (Table 2).

**Table 2:** Factors predictive of ileal intubation during screening colonoscopy based on multivariate analysis OR: Odds ratio, CI: Confidence interval

Variable	OR	Lower 95% CI	Upper 95% CI	p-value
Age	0.97	0.962	0.981	<0.001
Good quality of colon preparation	2.63	1.859	3.731	<0.001
Scope insertion time	0.93	0.907	0.954	<0.001
Training background (surgeon [reference group] vs. gastroenterologist)	4.52	1.013	20.154	0.048
Type of colonoscope (adult colonoscope [reference group] vs. pediatric colonoscope)	1.21	0.971	1.518	0.089
Morning procedures	1.08	0.893	1.312	0.422
Type of sedation (conscious sedation [reference group] vs. monitored anesthesia care)	1.15	0.731	1.809	0.547
Male gender	1.02	0.832	1.242	0.87

Among patients who underwent ileal intubation (group B), 37 (3.7%) were found to have a mucosal abnormality of the visualized ileal mucosa. The abnormal endoscopic ileal findings included ulceration (13), nodular/granular mucosa (nine), erosions (six), mucosal erythema (seven), inflamed mucosa (one), and ileal polyp (one). Endoscopic biopsies were obtained in 36 patients out of the 37 patients with abnormal ileoscopic examination. Histopathological evaluation revealed acute ileitis in 13 patients and chronic ileitis in five patients. The remainder were deemed non-pathological (normal ileal mucosa or prominent lymphoid hyperplasia). Therefore, the yield of clinically significant findings on histology was 1.8%.

## Discussion

Despite expert opinion recommending routine ileal intubation to preserve this endoscopic skill [8,9], the practice of ileal intubation during colonoscopy varies widely among endoscopists, ranging from 12%-96% [2,3,10-14]. Studies have reported that ileal examination is performed less often during SC than diagnostic colonoscopy performed for other indications. Utilizing the US national endoscopic database, comprising of 153,052 complete colonoscopies performed for various indications, Harewood et al. reported ileal intubation in 1800 of 25,249 average-risk SCs, accounting for an ileal intubation rate of 7.1% [14]. 

Variation in the reported rates of ileal intubation could be explained by the heterogeneity of the study populations, physicians' skills in performing ileal intubation, lack of guideline recommendations in this regard, the perceived low yield of routine ileal inspection in the setting of SC, and time constraints [4] 

The reported clinical yield of routine ileal inspection during colonoscopy varies, ranging from 1-4.6% [11,15-18]. This is even lower in asymptomatic patients undergoing screening or surveillance colonoscopy [2,15,18].

In our study cohort, routine ileoscopy revealed endoscopically abnormal mucosa in 3.7% of patients undergoing SC. However, in only around half of these cases (1.8% overall), the findings were deemed to be pathologically significant. This is in line with other reported studies in patients undergoing SC [2,15,18-20]. 

A study by Meral et al. looked at the diagnostic value of routine ileal intubation during colonoscopy in Turkey. Out of 1310 colonoscopies included in the study, 319 procedures were performed for screening purposes. Endoscopically abnormal findings of the terminal ileum were detected only in 11 patients (3.3%) of the screening cohort. The paper did not list the histopathological findings for the screening group. However, the study concluded that clinically significant histopathological findings were detected in only 22 cases (1.9%) among all patients who underwent colonoscopy with ileal inspection regardless of the indication [2].

Kennedy et al. reported their findings of terminal ileal intubation in 6408 patients who underwent screening colonoscopy in Mayo Clinic. Endoscopic abnormalities were identified in 1% of the cases. Histopathological abnormalities were detected in only 0.3% of this cohort [15].

Zwas et al. evaluated prospectively the diagnostic yield of ileoscopy in patients undergoing surveillance colonoscopy. They identified abnormal histopathological findings in 2.7% of the patients [18].

Geboes et al. compared 43 patients with a history of colon polyps to 257 patients with persistent diarrhea. The study did not detect any microscopic or macroscopic ileal lesions in the surveillance group. However, the authors reported microscopic ileal lesions in 49% of the patients with persistent diarrhea [20].

The main significant histopathological findings of the ileum in our study were acute and chronic ileitis. Interesting, ileitis was the most common histopathological finding in Meral et al. study (62.9%). In Kennedy et al.'s study, ileitis accounted for 50% of the histopathological findings, and the rest were labeled as normal mucosa or lymphoid hyperplasia [15].

Our study reported a higher ileal intubation rate among younger individuals undergoing screening colonoscopy. Existing studies report conflicting results regarding the association between the age of the patients and the likelihood of ileal intubation [2,14,21,22]. It is not clear why successful ileal intubation is more achievable in younger patients. One explanation could be related to the presence of more polyps in older subjects. More polyps burden may translate to a longer procedure time and that may deter endoscopists from attempting elective ileal intubation during SC for the sake of saving time.

In our cohort, poor preparation was associated negatively with the likelihood of successful ileal intubation. A prospective study conducted by Börsch et al. reached a similar conclusion [23]. Nonetheless, Buerger et al. suggested, in their retrospective study, that the quality of the colon preparation does not substantially impact the likelihood of ileal intubation [22].

Interestingly, the likelihood of ileal intubation during screening colonoscopy in our cohort was higher when the procedure was performed by a gastroenterologist compared to a surgeon (OR 4.52, p=0.048). We postulate that the difference may stem from variable attitudes among physicians regarding ileal intubation during SC.

Also, interestingly, short cecal insertion time was predictive of ileal intubation in our cohort. Lieman et al. reached a similar conclusion in their retrospective study that showed prolonged cecal insertion time was associated with a lower likelihood of ileal intubation (median and interquartile range: 6 [4,10] vs. 5 [4,8]; p<0.0001) [21].

Overall there was no significant difference in the total procedure time between the two groups in our cohort. Conflicting data exist in the current literature regarding the influence of terminal ileal intubation on the total procedure time of colonoscopy. Cherian et al. reported an increase in the total colonoscopy procedure time of 3 minutes due to ileoscopic examination [9]. In contrast, Leiman et al. reported no impact of attempting ileal intubation on the total procedure time [21].

The main limitation of the study is its retrospective design. Furthermore, the study population included patients age 40 and above. In the UAE, around 46% of colorectal cancers are detected in patients younger than 50 years of age [24]. Therefore, the current screening guidelines in the UAE recommend colonoscopy starting at the age of 40 for average-risk patients [25]. Hence, some of the results of this study may not be extrapolatable to countries where the average-risk screening population only comprises patients aged 50 or above.

## Conclusions

In average-risk individuals undergoing screening colonoscopy in the Middle East, the yield of endoscopic examination of the terminal ileum is low. Furthermore, the likelihood of detecting abnormal histopathological findings of the ileum of clinical significance in this setting is even lower. The findings of this study are consistent with the literature of the western population.

Because attempting ileal intubation is not associated with prolongation of the total procedure time, it is not unreasonable to perform ileal intubation to establish a complete colon examination during screening colonoscopy.
